# K/BxN Serum-Transfer Arthritis as a Model for Human Inflammatory Arthritis

**DOI:** 10.3389/fimmu.2016.00213

**Published:** 2016-06-02

**Authors:** Anne D. Christensen, Claus Haase, Andrew D. Cook, John A. Hamilton

**Affiliations:** ^1^Department of Medicine, University of Melbourne, Parkville, VIC, Australia; ^2^Novo Nordisk A/S, Måløv, Denmark

**Keywords:** K/BxN serum-transfer arthritis model, immune complex-driven arthritis, animal model, autoantibodies, rheumatoid arthritis

## Abstract

The K/BxN serum-transfer arthritis (STA) model is a murine model in which the immunological mechanisms occurring in rheumatoid arthritis (RA) and other arthritides can be studied. To induce K/BxN STA, serum from arthritic transgenic K/BxN mice is transferred to naive mice and manifestations of arthritis occur a few days later. The inflammatory response in the model is driven by autoantibodies against the ubiquitously expressed self-antigen, glucose-6-phosphate isomerase (G6PI), leading to the formation of immune complexes that drive the activation of different innate immune cells such as neutrophils, macrophages, and possibly mast cells. The pathogenesis further involves a range of immune mediators including cytokines, chemokines, complement factors, Toll-like receptors, Fc receptors, and integrins, as well as factors involved in pain and bone erosion. Hence, even though the K/BxN STA model mimics only the effector phase of RA, it still involves a wide range of relevant disease mediators. Additionally, as a murine model for arthritis, the K/BxN STA model has some obvious advantages. First, it has a rapid and robust onset of arthritis with 100% incidence in genetically identical animals. Second, it can be induced in a wide range of strain backgrounds and can therefore also be induced in gene-deficient strains to study the specific importance of disease mediators. Even though G6PI might not be an essential autoantigen, for example, in RA, the K/BxN STA model is a useful tool to understand how autoantibodies, in general, drive the progression of arthritis by interacting with downstream components of the innate immune system. Finally, the model has also proven useful as a model wherein arthritic pain can be studied. Taken together, these features make the K/BxN STA model a relevant one for RA, and it is a potentially valuable tool, especially for the preclinical screening of new therapeutic targets for RA and perhaps other forms of inflammatory arthritis. Here, we describe the molecular and cellular pathways in the development of K/BxN STA focusing on the recent advances in the understanding of the important mechanisms. Additionally, this review provides a comparison of the K/BxN STA model to some other arthritis models.

## Introduction

Research into the pathogenesis of rheumatoid arthritis (RA) has benefited enormously from a vast number of animal models, wherein mechanisms governing arthritis can be studied. These include both spontaneous models, such as the tumor necrosis factor (TNF) transgenic and interleukin-1 (IL-1) receptor antagonist-deficient mice, as well as induced models, most notably the collagen-induced arthritis (CIA) model.

In 1996, the K/BxN model of arthritis was reported for the first time by the Mathis/Benoist laboratory (1). This model was discovered by crossing T-cell receptor (TCR) transgenic KRN mice on a C57BL/6 background (transgenic for a TCR) recognizing a bovine ribonuclease peptide (RNase 43–56) presented by I-Ak major histocompatibility complex (MHC) class II molecule with autoimmune-prone non-obese diabetic (NOD) mice. Surprisingly, the F1-generation, called K/BxN mice, developed severe arthritis by the age of 4–5 weeks, which rapidly evolved until mobility was significantly suppressed (1). Arthritis progression in the K/BxN mice is driven by activation of T cells expressing the KRN TCR that recognizes a self-peptide bound to the NOD-derived I-A^g7^ molecule on MHC class II antigen-presenting cells (APCs). The peptide recognized by the K/BxN TCR, in the context of I-A^g7^, is the ubiquitously expressed self-antigen, glucose-6-phosphate isomerase (G6PI) (2), a cytosolic glycolytic enzyme catalyzing the inter-conversion of d-glucose-6-phosphate and d-fructose-6-phosphate (3). It was demonstrated that the activated T cells subsequently interact with B cells through TCR:A^g7^–MHC class II molecules and CD40:CD40L engagement, thereby promoting polyclonal B-cell activation and T-helper cell-dependent production of disease-inducing immunoglobulins (IgGs) (1, 3–5).

Importantly, it was further shown that transfer of purified IgGs or serum from arthritic K/BxN mice led to a robust and reproducible arthritis in many mouse strains, such as BALB/c, C57BL/6, and DBA/1 mice (6), as well as B-cell- and lymphocyte-deficient mice (4). Since transfer of K/BxN sera leads to reproducible disease in several mouse strains, it is an ideal model to study the effector mechanisms involved in progression of disease. As expected, arthritis induced by serum transfer is transient and wanes after 15–30 days but can be made persistent by repeated administration of antibody or serum (4). Overall, the discovery of K/BxN arthritis showed that joint-specific disease can be the consequence of systemic self-reactivity. The ability to transfer arthritis using pooled K/BxN sera has been widely used by many researchers to dissect several important effector pathways of arthritis *in vivo*. In the following sections, current knowledge about the K/BxN serum-transfer arthritis (STA) model is addressed, with focus on driver mechanisms responsible for progression of arthritis after serum transfer. Since B- and T-cells are not thought to play a major role in mediating arthritis after serum transfer they are not addressed in detail.

Since its discovery in 1996, the model has been used increasingly. From 2000 to 2005, only 15 papers were published about the K/BXN STA model (pubmed.com search under “KBxN serum transfer arthritis”); from 2005 to 2010, 53 papers were published, and from 2010 to 2015, the number was 87 with 18 papers published in 2015. Nonetheless, a thorough review of the literature about the model has not been published since 2004 (3). In addition to updating the literature on the K/BxN STA model, this review discusses the relevance of the model in relation to RA and provides a comparison to other RA models. It should also be noted that immune complexes (ICs) have been implicated in other arthritides, such as systemic lupus erythematosus (7) and ankylosing spondylitis (8); thus, though the focus on this review is on RA, it could also be relevant to other arthritides.

## The Initiation Phase of the K/BxN STA Model

**Figure 1:**
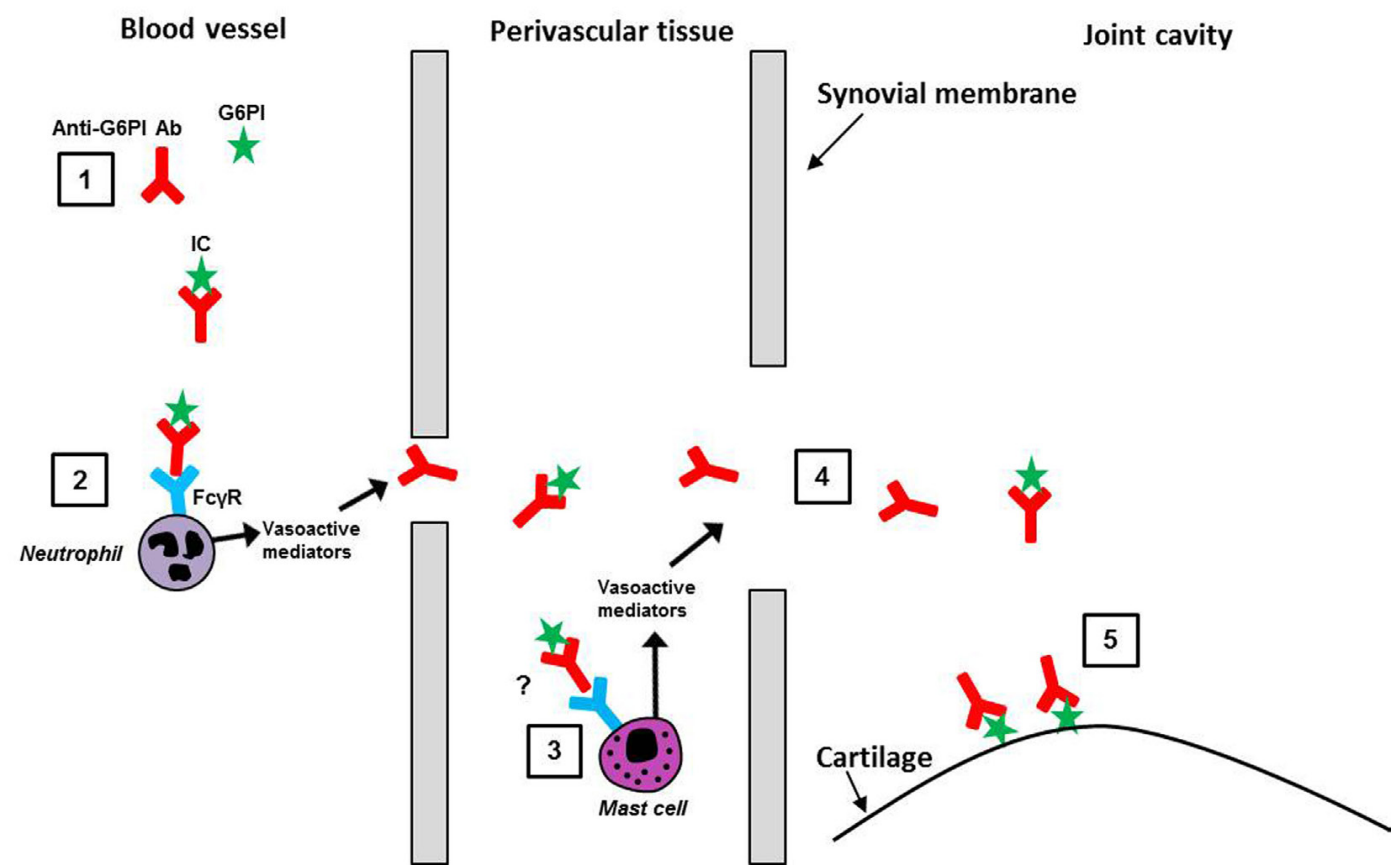
**The “initiation phase” of the K/BxN STA model**. A summary of some of the literature on the early stages of K/BxN STA progression, leading to the formation of anti-G6PI/G6PI ICs in the joint, is depicted. (1) In the blood, the anti-G6PI antibodies (Ab) bind to G6PI and form the ICs. (2) On neutrophils, for example, the ICs bind to Fcγ-receptors (FcγR) triggering the release of vasoactive mediators and the local increase in vascular permeability, thus allowing ICs and anti-G6PI Abs to enter the perivascular tissue in the joint. (3) In the perivascular tissue, the ICs might bind to FcγRs on mast cells causing them to degranulate, resulting in enhanced vascular permeability. (4) ICs, anti-G6PI Abs, non-specific Abs (not shown) and serum proteins (not shown) enter the joint cavity, where (5) anti-G6PI Abs bind to G6PI expressed on the cartilage surface.

The main features of what we have termed for convenience, albeit somewhat arbitrary, the “initiation phase” of the K/BxN STA model, are depicted in the literature-based schematic in Figure 1 and summarized below.

### Immune Complexes

**Figure 2:**
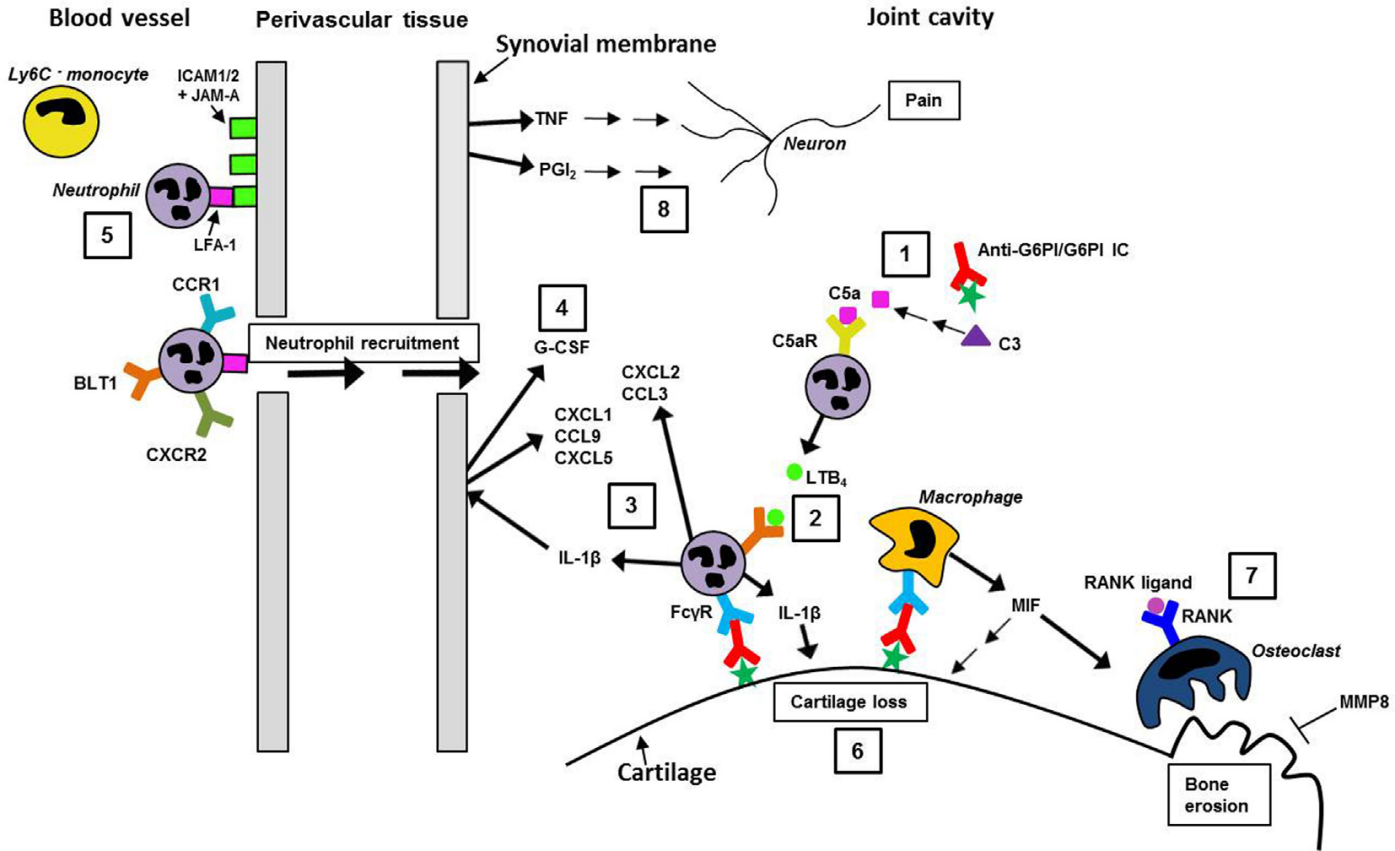
**The “effector phase” of the K/BxN STA model**. A summary of some of the literature on the subsequent stages of K/BxN STA progression, driven by joint-localized anti-G6PI/G6PI ICs and their proposed interaction with innate immune cells, such as neutrophils and macrophages, is depicted. (1) The alternative complement pathway is activated by the ICs, leading to C3 cleavage and eventually to the generation of C5a. Subsequently, C5a activates neutrophils *via* C5aR, which leads to their release of LTB_4_. (2) Activation of neutrophils by the LTB_4_/BLT1 interaction and (3) by Fcγ-receptors (FcγRs) leads to the release of interleukin 1β (IL-1β), which then induces neutrophil-attracting chemokines, for example, CXCL1, CXCL5, and CCL9, from resident tissue cells. Additionally, neutrophils participate in their own recruitment by releasing the chemokine CXCL2 (CXCR2 ligand) and to a lesser extent, CCL3 (CCR1 ligand). (4) IL-1β, and other pro-inflammatory cytokines, mediates the release of G-CSF locally in the joint, leading to neutrophil mobilization. (5) Leukocyte, for example, neutrophil, recruitment into the joint is facilitated by their LFA-1 binding to its ligands (ICAM1, ICAM2, and JAM-A) expressed on the activated vascular endothelium; Ly6C^−^ blood monocytes are also recruited (not shown). (6) Cartilage loss in the inflamed joint is mediated by, for example, IL-1β and macrophage-derived macrophage migration inhibitory factor (MIF). (7) Bone erosion upon osteoclast activation by RANK ligand/RANK interaction and release of MIF; MMP8 can protect against bone erosion and arthritis. (8) Both TNF and the prostaglandin, PGI_2_, are produced in the joint and either directly or indirectly mediate pain.

To address why the autoimmune inflammatory attack against the ubiquitously expressed enzyme, G6PI, occurs only in the distal joints, it was first shown that G6PI was present on the articular surface in ankle joints in normal mice and, most importantly, that its expression was amplified in arthritic mice (9). Second, it was demonstrated that purified anti-G6PI IgGs localized specifically to the distal joints in the front and rear limbs within minutes of intravenous injection, while control IgGs could not be detected in any joints. Binding of anti-G6PI IgGs reached saturation after 20 min and remained localized within the joints for at least 24 h (10). This led to the conclusion that anti-G6PI antibodies bind directly to extracellular G6PI in normal non-inflamed mouse joints, thereby forming anti-G6PI/G6PI ICs directly on the cartilage surface. The importance of these ICs was emphasized by the observation that pathogenicity after transfer of a pool of anti-G6PI monoclonal antibodies (mAb) was directly dependent on their ability to form mAb/G6PI ICs (11). Furthermore, it was demonstrated that soluble ICs, formed between anti-G6PI antibodies and G6PI in serum, played a central role in facilitating access for antibodies to the distal joints by binding to Fcγ-receptor (FcγR) III (12) possibly expressed on neutrophils in the blood (12, 13) (see also Fc Receptors below) but probably also on radio-resistant cells in an organ distant from the joint (14). It was further suggested that the activation of neutrophils subsequently triggers the release of vasoactive mediators, which leads to a local increase in vascular permeability, thus allowing ICs to enter the perivascular tissue in the joint and possibly bind to FCγRIII on mast cells found in close proximity to the microvasculature in the synovium (12). If this occurs, it would cause mast cells to degranulate, which results in an even larger and more widespread increase in the vascular permeability, thus allowing anti-G6PI antibodies, non-specific antibodies, and serum proteins to enter the joint. Once in the joint, the anti-G6PI antibodies bind to G6PI expressed on the articular cartilage surface and thereby mediate activation of different components of the innate immune system, for example, neutrophils (12) (discussed in Figure 2 below).

It is difficult to explain why ICs in serum facilitate access of antibodies exclusively to the distal joints, but it was suggested that arthritogenic antibodies cause an increase in the macromolecular vasopermeability in sites destined to develop arthritis (14). This increased vascular leakage in the distal joints mediated by anti-G6PI:G6PI ICs seemed to involve vasoactive amines, histamine, and serotonin. An increased vasopermeability was also induced by G6PI-non-specific ICs. However, the non-specific ICs did not promote arthritis development, indicating that simple access to the joint is not pathogenic, but there must be a targeting of ICs to some joint structures, for example, G6PI expressed on cartilage, to induce arthritis (14). Thus, it appears for reasons unknown that the distal extremities are especially prone to respond to systemic circulating ICs by vascular leakage, which subsequently facilitates access of arthritis-inducing antibodies into the joint.

### Fc Receptors

An important link between antibodies and activation of the immune system is Fc receptors. Four types of murine FcγR have been identified. FcγRI, FcγRIII, and FcγRIV mediate activating signals *via* the common γ-chain when cross-linked with ICs, while FcγRII inhibits cellular activation upon ligation (15). The role of the different FcγRs in the K/BxN STA model was explored using knockout (KO) mice. FcγR KO mice were protected from arthritis demonstrating the crucial role of FcγRs in this model (16). Lack of FcγRI had no influence on arthritis development (16), whereas FcγRIII-deficient mice developed reduced arthritis with delayed onset (13, 16, 17). The importance of FcγRIV was demonstrated by Mancardi et al. (18), who showed that blockade of FcγRIV with anti-FcγRIV mAb prevented development of arthritis. Additionally, it has been demonstrated that mice with a specific deletion of FcγRIV on osteoclasts were protected from arthritis induced by K/BxN serum suggesting an important role for FcγRIV in maturation and activation of osteoclasts and ultimately bone destruction (19). When addressing the impact of the inhibitory FcγRIIB, it was first observed that mice lacking this receptor developed arthritis similar to wild-type (WT) mice (6); however, three later studies demonstrated an enhanced disease progression in FcγRIIB KO mice compared to WT mice, indicating that this inhibitory Fc receptor has an immunosuppressive role in the model (13, 17, 20). In conclusion, these results reveal that in the K/BxN STA model FcγRIII is the dominant activating Fc receptor, FcγRIV is contributing to a minor extent, and FcγRII has an inhibitory role in disease development.

As an indication of the importance of Fc receptors in arthritis, intravenous immunoglobulin (IVIG) is widely used as a therapeutic strategy for suppression of autoantibody-triggered inflammation in a variety of clinical settings. The immunoglobulins are administered in a dose of 1–2 g/kg, and their anti-inflammatory effect has been proposed to be the result of several different mechanisms (21). In the K/BxN STA model, IVIG has been shown to suppress the progression of arthritis indicating that Fc receptors are important; however, the mechanisms of action have been the subject of disagreement (20–23). First, the mechanism behind the immunosuppression was reported to be induced by CSF-1-dependent macrophages, which by acting as “sensors” for the Fc fragments of IVIG, lead to the induction of FcγRIIB expression on CSF-1-independent “effector” macrophages at the site of inflammation, thereby raising the threshold for their activation by ICs (20). The immunoregulatory effect of IVIG has also been investigated in immune thrombocytopenia in mice and was shown, as in the K/BxN STA model, to result in an induction of FcγRIIB on splenic macrophages, thereby preventing FcγRIII-mediated clearance of IgG-opsonized platelets (24). Later, the anti-inflammatory effect was claimed to result from the presence of sialylated IgGs which are IgGs with sialic acid bound to glycan on their Fc region (23). These sialylated IgGs were shown to bind to the C-type lectin receptor SIGN-R1 on regulatory myeloid cells resulting in IL-33 release, which in turn stimulated IL-4 secretion from basophils; this IL-4 further promoted expression of the inhibitory Fc receptor, FcγRIIB, on effector macrophages (23). The same group further showed that IVIG or sialylated Fcs activated and expanded regulatory T cells in mice injected with K/BxN serum (22). However, the importance of sialylation has been questioned by the finding that removal of sialic acid residues by neuraminidase did not affect the suppression of K/BxN STA by IVIG (21); furthermore, the depletion of basophils did not abrogate the immunosuppression exerted by IVIG (21). The reason for the discrepancy between these results is currently unknown; however, it could be due to differences in, for example, the source of IVIG, route of administration, mouse strain, or experimental setup.

## The Effector Phase of the K/BxN STA Model

The essential link in the K/BxN STA model between antibody-dependent components, such as ICs, complement, and Fc receptors, and arthritis progression, is the activation of innate effector cells such as neutrophils, macrophages, and possibly mast cells. The main features of what we have termed for convenience the “effector phase” of the K/BxN STA model, involving the main effector cells and their responses, are depicted in the literature-based schematic in Figure 2 and are summarized below.

### Neutrophils

Neutrophils are readily activated by ICs and play an important role in the induction of arthritis in the K/BxN STA model. Their essential role was illustrated by Wipke et al. (25), who showed that mice depleted of neutrophils failed to develop arthritis – even neutrophil depletion after the onset of arthritis could reverse disease (25). This study used the neutrophil-depleting anti-Ly6G/C (Gr-1) mAb (clone RB6.8C5). However, the use of this antibody was recently challenged as it is thought to deplete not only LyG^+^ neutrophils but also a fraction of the Ly6C^+^ monocytes and macrophages (26). Specific depletion with anti-Ly6G mAb (clone 1A8) confirmed a role for neutrophils in the model and also showed that non-depleting doses of anti-Ly6G mAb (clone 1A8) attenuated progression of arthritis due to blockade of neutrophil migration into the joint (27). A crucial role of neutrophils was further supported by the observation that Gfi-1 KO mice, which have a selective defect in their ability to generate mature neutrophils, were resistant to arthritis (28).

To further understand the role of neutrophils and how they contribute to the induction of arthritis, different neutrophil-related effector mechanisms have been investigated, including the neutrophil-derived leukotriene B4 (LTB_4_) and its receptor, BLT1. LTB_4_ is a highly potent lipid chemoattractant produced by cells of the innate immune system (for example, neutrophils, macrophages, and mast cells). BLT1 is expressed on neutrophils and induces chemotaxis and adhesion in response to LTB_4_ (29, 30). It appeared that the arthritis progression in the K/BxN STA model was critically dependent on the generation of neutrophil-derived LTB_4_ (31), while neutrophil expression of BLT1 was required for arthritis generation as well as chemokine production, suggesting that neutrophils recruit other neutrophils in an autocrine manner (32). On further examination, BLT1-expression on neutrophils was found crucial for their release of the pro-inflammatory cytokine, IL-1β, into the joint which induced the production of neutrophil-active chemokines, for example, CXCL1, CXCL5, and CCL9, from resident tissue cells of the joint (33) (the role and impact of IL-1 β in the progression of arthritis in the K/BxN STA model will be discussed in further detail later). Moreover, neutrophils could directly participate in their own recruitment by expressing the CXCR2 ligand, CXCL2, and to a lesser extent the CCR1 ligand, CCL3 (33). Recruitment of neutrophils was demonstrated to be dependent on their expression of CXCR2 and partly of CCR1 (33, 34). Recently, the link between release of LTB_4_ and IL-1β from neutrophils and the requirement for C5a receptor (C5aR) and FcγR was addressed. It was demonstrated that C5aR- and FcγR activation on neutrophils were necessary for the initiation and progression of arthritis, and that C5aR activation of neutrophils was required for their LTB_4_ release, while FcγR engagement mediated IL-1β release (35) (complement activation is discussed below and in Figure 2). Activation of the two receptors occurred independently from each other without any cross-regulation between the two receptor classes. This is a unique observation compared to other models of autoantibody-induced inflammation where C5aR signaling is thought to set the threshold for subsequent sustained activation of FcγRs on resident tissue immune cells (36). The importance of neutrophil expression of FcγR and C5aR as well as their release of LTB4 and IL-1β in the K/BxN STA model was confirmed by Monach et al. (28). Furthermore, it was shown that C5aR and FcγR on mast cells did not contribute significantly to arthritis (35). Some of these neutrophil-dependent cascades are depicted in Figure 2.

That FcγR-expression on neutrophils is of crucial importance for the recognition of ICs was further addressed through the use of the spleen tyrosine kinase (Syk)-deficient mouse strain. Syk is required for signaling through FcγRs, integrins, and other scavenger receptors by using ITAMs to initiate intracellular signaling. First, it was shown that mice lacking Syk in all hematopoietic lineages were resistant to disease in the K/BxN STA model (37). Second, specific deletion of Syk in neutrophils was sufficient to block the initiation of arthritis, which emphasizes the importance of FcγR signaling in neutrophils after engagement with ICs (38). Furthermore, we have recently shown that granulocyte colony-stimulating factor (G-CSF), possibly produced from IL-1β activation of a resident cell population, is a pivotal driver of the disease progression in the K/BxN STA model and possibly acts, in part, by regulating neutrophil numbers in the circulation (39) (Figure 2).

In conclusion, it is evident that neutrophils are a pivotal cell type in the K/BxN STA model and play an essential role in progression of arthritis. In addition, release of LTB_4_ and IL-1β amplifies arthritis by driving the ongoing recruitment of neutrophils.

### Macrophages

Macrophages are present in high numbers in the inflamed joint tissue in RA, and activation of macrophage FcγRs by ICs at the site of inflammation has been demonstrated to be crucial in the pathogenesis of an immune complex-induced arthritis (ICA) model (40). In the K/BxN STA model, the importance of macrophages has been demonstrated by depletion studies showing that a lack of macrophages resulted in complete resistance to arthritis (41). Importantly, subsequent studies showed that arthritis could be induced in macrophage-depleted mice by reconstitution with peritoneal macrophages, hence confirming a key role for macrophages (41). However, it has also been shown that K/BxN serum-induced arthritis is independent of CSF-1-dependent macrophages since op/op mice, which lack CSF-1, were fully susceptible to arthritis (20). This suggests that CSF-1-independent macrophages are the effector macrophages in this model. Moreover, a recent study by Misharin et al. (42) demonstrated that non-classical Ly6C^−^ blood monocytes were recruited to the joint during inflammation in the K/BxN STA model where they gave rise to inflammatory macrophages, a subtype known as M1 macrophages, and were shown to be crucial for development of arthritis (42). Additionally, they showed that tissue-resident macrophages in the joint played an important role in maintaining joint integrity and resolution of inflammation and that they thereby belong to the M2 subtype of macrophages (42). Interestingly, their data further suggested that the recruited macrophages initially expressed a complex set of both M1 and M2 genes followed by a shift toward a more M2 phenotype where they, together with the tissue-resident macrophages, attenuated the severity of arthritis (42). Taken together, these data suggest that circulating Ly6C^−^ non-classical monocytes are recruited to the joint in the initiation phase of arthritis, where they orchestrate both the development and resolution of the joint inflammation.

The role of granulocyte–macrophage colony-stimulating factor (GM-CSF) has also been examined in the K/BxN STA model (43). GM-CSF is considered to be an important differentiation/activation factor for macrophages and granulocytes and is also a pro-inflammatory mediator (43), with encouraging RA trials targeting it or its receptor having been completed (44). Its importance in arthritis progression was illustrated by the observation that in the K/BxN STA model, both GM-CSF KO mice and WT mice treated with anti-GM-CSF mAb showed less severe arthritis but with a similar time of onset compared to WT control mice (43). This was suggested to be a consequence of a reduced number of monocytes in the circulation and a decline in the number of synovial macrophages. The data indicated that in the absence of GM-CSF, the early neutrophil-mediated response was still present, whereas later, the macrophage-driven response was defective (43). Another aspect of the role of macrophages in the K/BxN STA model has been revealed through studying the effect of IVIG treatment as described previously (20). It was shown that IVIG protected against arthritis induced by K/BxN serum through a macrophage-specific induction of FcγRIIB expression at the site of inflammation, which raised the threshold for their activation by ICs (20).

Taken together, these results suggest that macrophages constitute an important cell type in the K/BxN STA model.

### Mast Cells

The role of mast cells in the K/BxN STA model has been controversial mainly due to the different KO strains used in the various studies. Insights into the role of mast cells in many inflammatory responses relies predominately on a mouse strain deficient in mast cells due to a mutation in the gene encoding the receptor tyrosine kinase Kit, referred to as the white-spotted (W) Kit allele (45). The K/BxN STA model was initially induced in two versions of this strain, the mast cell-deficient Kit*^*W/W-v*^* strain and the mast cell-deficient Kit-ligand (Kitl)-mutated Kitl^Sl/Sl-d^ strain (46). Both strains displayed little or no clinical or histologic signs of arthritis, and transfer of mast cells into both strains restored their ability to develop arthritis (46). It was also shown that mast cells in the initiation phase of the disease were activated through FcγRIII, which led to release of IL-1 (47), and arthritis could be suppressed by preventing the activation-induced degranulation of mast cells by treatment with the cAMP-inducing agent, salbutamol (48). Another way that mast cells might drive arthritis was proposed to be through the release of tryptase/heparin complexes, which were shown to induce the expression of the neutrophil chemoattractants, CXCL1, CXCL5, and CXCL8, in cultured fibroblast-like synoviocytes (49). Recently, the use of the mast-cell-deficient Kit^W/W-v^-strain has been questioned, since it also shows defects in many other cell lineages, such as red blood cells and neutrophils (45). When K/BxN STA arthritis in the Kit^W/W-v^-strain was compared with another mast cell-deficient strain, the Kit^W-Sh^-strain, it was found that arthritis did not develop in the Kit^W/W-v^-strain but that the Kit^W-sh^-strain was fully susceptible. This was explained by the neutropenia found in the Kit^W/W-v^-strain but not in the Kit^W-sh^-strain (18, 38). A dispensable role for mast cells was confirmed by the use of a non-kit mutated mast-cell-deficient mouse strain, the *Cpa3*^cre/+^-mouse, which developed arthritis and, besides a lack of mast cells, display a normal immune system (45). The discrepancy between arthritis-susceptible *Cpa3*^cre/+^-mice and arthritis-resistant Kit^W/W-v^-mice suggests an important role for Kit in this model. Also, since transfer of mast cells rendered Kit^W/W-v^-mice susceptible to arthritis (46), it is possible that mast cells have pro-pathogenic effects when Kit is missing. In combination, these experiments suggest that the previous conclusion regarding the importance of mast cells in the K/BxN STA model could be based on a misinterpretation of the lack of arthritis in the Kit^W/W-v^-strain. Mast cells might contribute, but based on the recent study in the mast cell-deficient *Cpa3*^cre/+^-strain (45), they appear dispensable.

### Natural Killer T Cells

A role for natural killer (NK) T cells in the K/BxN STA model was suggested by the observation that arthritis was attenuated in CD1d KO and Jα281 KO mice, both deficient in NKT cells (50). The mechanism was thought to involve NKT-mediated suppression of transforming growth factor β1 (TGF-β1) production in the joint tissue, which in turn was dependent on release of IL-4 and interferon γ (IFNγ) from the NKT cells (51). Furthermore, it was shown that CD1d KO mice exhibited less severe arthritis and that arthritis could only be restored with transfer of NKT cells from WT mice and not with transfer of cells from FcγR KO mice. These findings indicate that binding of IgG to FcγRIII on NKT cells in the joint induces their activation and participation in the induction of arthritis (50).

### Complement

Development of arthritis in the K/BxN STA model involves activation of the complement cascade. It is believed that the alternative pathway is important, since mice lacking complement factor B did not develop arthritis (16). The classical pathway or the mannose-binding pathway is not thought to be relevant, since transfer of K/BxN sera to mice deficient in C1q, C4, and mannose-binding-protein A (MBP-A) resulted in normal arthritis development (16). This conclusion was further supported by Solomon et al. (52) who showed that mice deficient in C4 or the complement receptors 1 and 2, all components of the classical pathway, were susceptible to K/BxN STA (52). All three pathways of the complement system lead to the generation of C3, which has been shown to be indispensable for arthritis induction, thus emphasizing the general importance of complement activation in this model (16, 53); in contrast, the C3-receptor seems to be dispensable (16). C3 deposits have been found locally in the arthritic joints (9, 16), and it has been suggested that during the inflammatory response, C3 is produced by parenchymal cells distant from the joint and transported to the joints *via* the circulation (53). In addition, it has been shown that circulating C3 is necessary and sufficient for arthritis induction (53). Downstream of C3, the complement cascade, leads to activation of C5 that is a central mediator in the complement network. In the joints of the K/BxN STA model, the alternative complement pathway is activated by the anti-G6PI/G6PI ICs, leading to C3 cleavage and generation of C5 (Figure 2). The importance of C5 in the K/BxN STA model was demonstrated by the fact that C5 KO mice showed no signs of disease (16). This was further confirmed by blockade of C5 with anti-C5 mAb, which both prevented disease by treatment prior to onset and reversed ongoing disease when injected several days after arthritis onset (16). C5 is cleaved by C5 convertase into the soluble component, C5a, and the C5b fragment that remains bound to the cell surface and initiates the formation of the membrane attack complex, the final step of complement activation. Formation of the membrane attack complex is not required in the K/BxN STA model since C6 deficiency (a component of the complex) did not influence disease progression (16). In contrast, C5a is crucial for the development of arthritis illustrated by the fact that C5a–C5aR KO mice were completely resistant to arthritis (16). C5a functions as a chemoattractant and inducer of acute inflammation, for example, by activating neutrophils and mast cells, stimulating endothelial cells to express P-selectin and increasing vascular permeability (3). Since antibodies generally activate complement through C1q and the classical pathway, it was a surprise that this pathway was not involved in driving the disease. However, the dominant isotype in anti-G6PI antibodies is the IgG1-subclass (11) and, since mouse IgG1 is a weak activator of C1q, a component of the classical pathway (54), it may explain why the alternative pathway is the one important for arthritis induction in the K/BxN STA model. Additionally, multimerized IgG1 is efficient in activating FcγRIII, which is also an important driver of the inflammatory response in the model, as described above (11).

### Pro-inflammatory Mediators

Pro-inflammatory cytokines, such as TNF, IL-1, and IL-6, are pathogenic drivers in both RA and in many mouse models of arthritis; however, the impact of each cytokine on the disease progression can differ from patient to patient (55) and from model to model (56). In the K/BxN STA model, studies have indicated that TNF, IL-1β, and IL-6 were released in the inflamed joint (57), but a role for TNF could not initially be proven (58). Subsequently, the significance of different selected cytokines was studied in further detail by Ji et al. (57) who induced the K/BxN STA in a panel of mouse strains deficient in one or more inflammatory cytokines or their receptors. These results demonstrated a critical role for IL-1, since no clinical signs of disease were observed after transfer of K/BxN serum into IL-1R-deficient mice, in which both IL-1α- and IL-1β-mediated signaling are blocked. In relation to IL-1, it should also be mentioned that IL-1 receptor antagonist, which is a well-known anti-inflammatory mediator, controlled articular inflammation during the acute phase of K/BxN STA (59). The results obtained for TNF involvement in the model were mixed, since arthritis developed in one-third of the TNF-deficient mice, indicating that TNF is an important, but dispensable, driver of disease development (57). In contrast to TNF and IL-1, IL-6 did not play a role in driving the inflammatory response (57). This suggests that in the K/BxN STA model, IL-1 signaling is crucial (Figure 2), TNF plays an important but partial, role, and IL-6 is completely dispensable.

The recently discovered IL-17 family of cytokines, especially IL-17A and IL-17F, which are the most homologous members of the family, has been shown to be implicated in the pathogenesis of many autoimmune diseases including RA (60, 61). They have also been investigated in the K/BxN STA model. First, it was shown that administration of anti-IL-17A mAb did not exert any effect on arthritis progression (62). Later, it was found that mice deficient in IL-17 receptor subunit A were protected from the serum-induced arthritis, while recently, it was further demonstrated that IL-17A KO mice exhibited reduced arthritis (63). The same study additionally showed that neutrophils are an essential source of IL-17 in the model (63). It has further been found that local administration of IL-17 *via* intra-articular injection of an adenovirus vector increased the severity of arthritis in the K/BxN STA model (64). The discrepancy between the anti-IL-17 mAb study and mice deficient in IL-17A or IL-17 receptor subunit A might be explained by insufficient antibody dosing or incomplete blocking of IL-17 arising from the tight interaction between effector cells.

In addition to increased levels of pro-inflammatory cytokines, high levels of prostaglandins (PGs) are also found in RA patients. They are lipid mediators that, besides having a role in many physiologic activities, also play an important role in pathologic inflammation (65). In the K/BxN STA model, high levels of PGE_2_, as well as the stable metabolite of PGI_2_, 6-keto-PGF1α, were found in inflamed joints, although only PGI_2_ appeared to be indispensable for arthritis development (65) (Figure 2). Furthermore, it was demonstrated that arthritis was reduced by pharmacological inhibition of PG synthesis by administration of a potent inhibitor of both cyclooxygenase (COX) 1 and 2. Interestingly, it was additionally shown that even though both COX isoforms were found in the inflamed joints in the model, only COX1 contributed substantially to disease (65). Inhibition of COX1 and/or COX2 is the mechanism of action behind the immunosuppressive effect of non-steroidal anti-inflammatory drugs (NSAIDs) that have been used frequently for amelioration of symptoms in RA patients (66).

### Toll-Like Receptors

Toll-like receptors (TLRs) are a group of receptors that serve to recognize pathogen-associated molecular patterns (PAMPs), which often comprise microbial products such as lipopolysaccharide (LPS) and peptidoglycans. When one of these ligands binds to TLRs on cells of the innate immune system, it leads to a range of responses, including the release of different cytokines and activation of APCs (67). The TLRs differ in their extracellular structure from the receptors for IL-1 and IL-18; however, they share the same intracellular signaling pathway, which includes myeloid differentiation factor 88 (MyD88) (68). In the K/BxN STA model, the role of TLR4 was first investigated. It was shown that arthritis progression in TLR4 KO mice had a similar initiation phase but was not sustained compared to WT mice, suggesting that TLR4 plays a role in the later phase in the model (69). Additionally, the same study showed that MyD88 is a key molecule and confirmed the essential role of IL-1 since no arthritis was observed in either MyD88- or IL-1R KO mice (69). Interestingly, LPS, a TLR4 ligand, circumvented the requirement for IL-1R signaling (69). However, recent studies reported that TLR4 KO mice exhibited either a similar progression of arthritis (70, 71) or a reduced arthritis even in the early induction phase (72) compared to WT mice. These discrepancies demonstrate that the impact of TLR4 on arthritis development in the K/BxN STA model varies from study to study and that further studies are therefore needed to clarify its role.

The role of another TLR and its ligands in the K/BxN STA model has been investigated by Wu et al. (73) who, surprisingly, discovered that certain unmethylated DNA CpG motifs (CpGs), with their signaling being mediated through TLR9, were able to inhibit arthritis. This inhibition relied on cells of the innate immune system and, specifically, on crosstalk between dendritic cells (DCs) and NK cells. It was suggested that CpGs bind to DCs *via* TLR9, which in return release IL-12 that further stimulates NK cells to produce IFNγ. IFNγ subsequently inhibits neutrophil migration into the joint (73). These observations describe a potential anti-inflammatory role for both CpGs and IFNγ in this model underlining the complexity of the innate effector mechanisms driving the pathogenesis. Additionally, an inhibitory role has been suggested for TLR2, which was shown to regulate arthritis in the model by controlling the inhibitory FcγRIIB on macrophages (71). Finally, a recent study showed that both TLR3 KO and TLR7 KO mice developed reduced K/BxN serum-induced arthritis (74) and that the transcription factor, interferon regulatory factor 5 (IRF5), contributed to disease progression by mediating pro-inflammatory cytokine production, mainly that of IL-1β generated downstream of TLR3 and TLR7 (74).

Taken together, these studies suggest that TLR3, TLR7, and possibly TLR4 play a pro-inflammatory role in the K/BxN STA model, while TLR9 and TLR2 mediate an anti-inflammatory effect.

## Integrins

Leukocyte infiltration into the synovial tissue is a crucial step in the pathogenesis of inflammatory arthritis and involves a range of different integrins and their ligands. The β_2_ integrins are heterodimeric cell proteins consisting of a common β-chain (CD18) that pairs with one of four α-chains: α_L_ (CD11a), α_M_ (CD11b), α_X_ (CD11c), or α_D_ (CD11d). Ligands for CD11a/CD18 (LFA-1) include ICAM-1, -2, -3, and junctional adhesion molecule (JAM)-A (75), while ICAM-1 and iC3b are ligands for CD11b/CD18 (Mac-1, CR3) (76). In the K/BxN STA model, it was demonstrated that β_2_-deficient mice were resistant to arthritis and that CD11a α-chain (LFA-1) was critical for this process (77). Moreover, blockade of LFA-1 with an anti-LFA-1 mAb reduced the already ongoing inflammation, and functional blockade of its counter-receptors, ICAM-1, ICAM-2, and JAM-A, provided a reduction, but not complete amelioration, of arthritis (77). It was also shown that ICAM-1, ICAM-2, and JAM-A were mainly expressed by the vascular endothelium, while LFA-1 was expressed on leukocytes within the synovial lining and within the inflammatory synovial fluid (77). This study did not investigate which subtype of leukocytes expressed LFA-1; however, another study subsequently demonstrated that LFA-1 expression on neutrophils was crucial for arthritis development in the K/BxN STA model (28). It is reasonable to consider that LFA-1 expression on monocytes/macrophages might also contribute to arthritis progression. In this context, LFA-1 was shown not to be important for monocyte migration in an acute murine peritonitis model, suggesting that LFA-1 does not play a role in infiltration of monocytes into inflamed tissue (78). However, whether this is the same for the K/BxN STA model should be examined.

In conclusion, recruitment of leukocytes into the joints and the ensuing arthritis in the K/BxN STA model seem to be dependent on the expression of LFA-1 on leukocytes, especially neutrophils, and the binding to its ligands, ICAM-1, ICAM-2, and JAM-A, expressed on the activated vascular endothelium (Figure 2).

## Disease Manifestations

### Cartilage Loss and Bone Erosion

Cartilage loss is a well-studied pathological manifestation of arthritis in the K/BxN STA model (57, 79). IL-1β is often a crucial mediator of cartilage destruction in arthritis models (80), and after induction of K/BxN STA, IL-1R KO mice showed, in addition to complete arthritis suppression, no signs of cartilage destruction (57), suggesting that IL-1β is a crucial driver of cartilage destruction also in this model. Additionally, bone erosion, *via* osteoclast activation in inflamed joints, is involved in the pathogenesis of RA and has also been studied in the K/BxN STA model. One of the essential factors for osteoclast differentiation and activation is the receptor activator of nuclear factor-κβ ligand (RANKL), and it was shown that RANKL KO mice were protected from bone erosion after transfer of K/BxN serum (81) (Figure 2). In contrast, a deficiency in matrix metalloproteinase-8 (MMP8) increased joint inflammation and bone erosion in the model, suggesting that MMP8 protects against the inflammatory synovitis and bone erosion (79). A cytokine that could also play a role in RA is macrophage migration inhibitory factor (MIF) (82). It was first demonstrated that this cytokine was essential for disease development in the K/BxN STA model since both joint inflammation and cartilage destruction were significantly reduced in MIF KO mice (83) (Figure 2). Furthermore, the same study demonstrated that adoptive transfer of WT macrophages could restore the sensitivity of MIF KO mice to arthritis development (83). Recently, it was further demonstrated that MIF KO mice transferred with arthritic K/BxN serum, in addition to reduced joint inflammation, exhibited markedly reduced bone erosion compared to WT mice. It was also shown *in vitro* that MIF facilitated RANKL-induced osteoclastogenesis, which suggests that MIF contributes directly to bone erosion as well as to inflammation in arthritis (84) (Figure 2). Taken together, these studies suggest that the K/BxN STA model can also be used as an animal model, wherein the different mechanisms behind bone erosion can be studied in addition to those governing cartilage loss.

## Pain

Recently, an increasing focus has been on the interface between the immune and nervous systems in inflammatory pain, with pain being a clinical symptom with high negative impact on patients suffering from different autoimmune diseases and especially RA. Pain in RA is difficult to control and often persists after resolution of joint swelling with anti-inflammatory treatments (85). To address the unmet need for pain relief, inflammatory pain has lately been investigated in several arthritis models including the K/BxN STA model (86, 87). Christianson et al. (86) showed that arthritis in the K/BxN STA model led to persistent pain with mechanical hypersensitivity not returning to baseline and outlasting the inflammation by 2 weeks (86). Moreover, the pain was shown in the inflammatory phase to be sensitive to treatment with NSAIDs and etanercept (TNF blocker) (Figure 2), and this was not the case in the post-inflammatory phase (86). Furthermore, the same group showed that during the persistent pain seen after resolution of inflammation, spinal TLR4 played an important role (70).

An interesting aspect of pain is the involvement of capsaicin-sensitive neurons that not only can mediate pain but also contribute to inflammatory processes by release of neuropeptides such as substance P and calcitonin-gene-related peptide (CGRP) (88). In the K/BxN STA model, it was demonstrated that in the early phase, capsaicin-sensitive neurons played an anti-inflammatory role, while in the later phase (10 days post-serum injection), they contributed to mechanical hyperalgesia (88). Furthermore, the neuropeptide, neuromedin U, which has pro-inflammatory activity, was shown to promote arthritis in the K/BxN STA model (89). The effect of denervation on arthritis progression has also been explored in the K/BxN STA model. Interestingly, it was found that denervation of one limb prior to the serum transfer protected that limb from arthritis *via* an impact on the microvasculature. The joint-localized vascular leak that normally causes swelling and edema in the affected joint was compromised in the denervated limbs (90). This finding underlines the fact that arthritis, even in a rather simple model system as the K/BxN STA model, results from complex interactions between the nervous, immune, and vascular systems.

Overall, these recent studies within pain suggest that the K/BxN STA model is useful for the exploration of pain mechanisms and could be a potential model to explore novel pain-modulating treatment strategies for RA.

## A Comparison of the K/BxN STA Model with Other Arthritis Models

### Collagen Antibody-Induced Arthritis Model

Among the many different mouse models of arthritis, the K/BxN STA model is most similar to the collagen antibody-induced arthritis (CAIA) model. The main similarities and differences between the features of the CAIA and K/BxN STA models are listed in Table 1. In the former model, arthritis develops after administration of a defined cocktail of anti-collagen type II mAb (anti-CII mAb), most often together with LPS (91). When LPS is administered with the antibody cocktail, it enhances the incidence and severity of the disease and thereby reduces the amount of mAb required to induce arthritis. Both models represent the effector phase of arthritis, work in many mouse strains, and have a similar time scale. Both the CAIA and K/BxN STA models are driven by antibodies and the formation of ICs (91), and both can be induced in T cell- and B cell-deficient mice (4, 92, 93). In both models, ICs initiate the inflammatory response either *via* activation of the complement system or by direct engagement and activation of Fc receptor-bearing immune cells (91). As to the role of Fc receptors, the two models also share similar dependencies since CAIA mice lacking the general FcγR-chain were highly resistant to arthritis, but FcγRIII-deficient mice were only partially resistant (94), an observation also seen in the K/BxN STA model (16). Additionally, complement activation and the downstream effects of C5a and C5aR are key factors in the pathogenesis of arthritis in both models (16, 95, 96). As in the K/BxN STA model (16), the alternative complement activation pathway has been suggested to be the dominant pathway for inflammation and joint destruction in the CAIA model (97–99). However, in the CAIA model, the classical pathway additionally seemed to participate in complement activation, which indicates that both pathways might play a role (98), an observation different from that in the K/BxN model. This difference could be explained by the fact that IgG1 is the dominant isotype of the anti-G6PI antibodies (11), whereas IgG2a and IgG2b are the most widely used for the induction of CAIA (93). Murine IgG2a, IgG2b, and IgG3 isotypes have been shown to activate the classical pathway, whereas IgG1 has been suggested to activate the alternative pathway (54).

**Table 1:** **Similarities and differences between the K/BxN STA, CAIA, and CIA models**.

Feature	K/BxN STA model	CAIA model	CIA model
Phase(s)	Effector	Effector	Immunization and effector
Susceptible strains	Multiple strains (6)	Multiple strains (100)	DBA1, B10Q, B10.QRIII, and C57BL/6 (less severe) (101)
Time to termination	10–15 days normally (102)	10–15 days normally (91)	5–6 weeks (101)
Immunization (involvement of T- and B-cells)	No (4)	No (93)	Yes (101)
Antigen	G6PI (2)	Collagen II (91)	Collagen II (101)
Immunostimulatory components	None (102)	LPS (91)	CFA (103)
Autoantibodies	Main drivers – ICs formed systemically and in the joint (9)	Main drivers – ICs formed in the joint (12)	Yes (104)
Isotype of autoantibodies	IgG1 (11)	• IgG2a • IgG2b (93)	IgG2a (104)
Fc receptors	• FcγR • FcγRIII • FcγRIIB (16)	• FcγR • FcγRIII (94)	• FcγR (105) • FcγRIII (106) • FcγRII (105)
Complement system	• C5aR (16) • Alternative pathway (16)	• C5aR (95) • Alternative and classical pathways (98)	• C5aR (107) • Alternative and classical pathways (108)
Important immune cells	• Neutrophils (25) • Macrophages (41) • Mast cells? (45, 46)	• Neutrophils (96)	• Neutrophils (109) • Macrophages (110) • T cells (101) • B cells (101)
Pro-inflammatory cytokines	• TNF (57) • IL-1β (57) • G-CSF (39) • GM-CSF (43) • MIF (83, 84) • IL-17? (62, 63)	• TNF (92) • IL-1β (92) • IL-4 (111)	• TNF (80) • IL-1β (80) • G-CSF (109) • GM-CSF (112) • IL-6 (113) • IL-17 (114)

As in the K/BxN STA model, neutrophils play an essential role in the CAIA model and were indispensable both for the development and maintenance of arthritis (96). With regard to effector cytokines, both TNF and IL-1β have been shown to be the major effector cytokines in CAIA, and blockade of either of these cytokines ameliorated the disease, whereas IL-6 blockade had no effect (92). Hence, TNF seems to be more crucial in the CAIA model than in the K/BxN model where, as mentioned, divergent results were obtained after neutralizing TNF (57). Another cytokine that has been shown to play different roles in the two models is IL-4. It was reported to be an important cytokine in the CAIA model, and its neutralization greatly reduced the severity of the disease (111), whereas IL-4 was shown to play no role in the K/BxN model (115).

The most significant difference between the two models is the specificity of the antibodies driving the disease. In the CAIA model, the antibodies are anti-CII mAb (91), whereas the K/BxN STA model is driven by arthritogenic serum containing polyclonal antibodies directed toward G6PI (3). This difference, among other aspects, leads to different localization of the respective antibodies in the joints. Both types of antibodies bind to the cartilage surface (10, 116); however, the anti-CII mAb also penetrate the cartilage (117). Anti-G6PI serum is more effective at inducing arthritis than anti-CII mAb as illustrated by the fact that the K/BxN STA model does not need the co-injection of LPS as the CAIA model does, and a lower dose of purified anti-G6PI IgGs is required to induce arthritis compared to the dose of anti-CII mAb routinely used (11).

Another important difference between the two models is the way the antibodies enter the joints. In the K/BxN STA model, ICs are formed systemically due to the fact that G6PI is ubiquitously present (9) (Figure 1). As mentioned, the systemic ICs activate inflammatory cells, such as neutrophils, which subsequently release mediators that increase the vascular permeability, thereby giving ICs and antibodies access to the joint (Figure 1). In CAIA model, the collagen II (CII) antigen is mainly present in the joint, and ICs cannot be formed systemically (12), which means that the anti-CII mAb given systemically do not readily localize in the joints without any additional trigger. Therefore, LPS or a high dose of antibody is needed to ensure that the anti-CII mAb enter the joints (12). Overall, despite many similarities between the two models, differences also exist such that each model can be used to inform on different aspects of arthritis progression.

### Collagen-Induced Arthritis Model

The CIA model is one of the most widely used animal models for RA and shares many similarities with it. It is, therefore, relevant to highlight the similarities and differences between the CIA and K/BxN STA models and the advantages of each model. The main similarities and differences between the features of the CIA and K/BxN STA models are also listed in Table 1. The CIA model is induced by immunization of susceptible mouse strains, such as DBA/1, B10.Q, and B.10RIII, with CII in complete Freund’s adjuvant (CFA) (103). However, it can also be established in C57BL/6 mice, thereby increasing the range of KO mice that can be studied, although the incidence and severity are less than in the DBA/1 or B10.RIII strains (101). Typically, chicken or bovine CII is used. The mice can be boosted with CII emulsified in incomplete Freund’s adjuvant (IFA) 3 weeks later; however, this is usually only necessary when using specific types of collagen such as α1(II) chains or purified cyanogen bromide fragments of CII (101). Additionally, occasionally LPS is given around the time of the boost to induce the disease to occur more rapidly and with less variability in onset (118). Normally, arthritis appears 3–5 weeks after immunization and usually peaks by week 6 (101). It presents as a polyarthritis most prominently in the limbs and characterized by inflammatory synovial infiltration, cartilage and bone erosion, and synovial hyperplasia similar to RA (119). Generally, susceptibility has been linked to strains that have MHC class II I-A^q^ and I-A^r^ haplotypes; however, it is evident that many mouse strains have variable degrees of susceptibility to CIA (119). The biggest difference between K/BxN STA and CIA is of course that CIA is the result of an active immunization, while arthritis in the K/BxN STA model is induced by passive transfer of autoantibodies. Since an immunization takes place in the induction phase of CIA, arthritis in this model is dependent on stimulation of collagen-specific T cells and the production of high titers of autoantibodies specific for CII by B cells (101). As mentioned, in the K/BxN STA model, both T cells and B cells are dispensable (4). However, similar to what is the case in the K/BxN STA model (25, 41), both neutrophils (109) and macrophages (110) have been shown to be important cell types in the CIA model. With regard to the role of Fc receptors, the two models show similar dependencies since mice lacking the general FcγR-chain were highly resistant to CIA (105) and FcγRIII-deficient mice were partially resistant (106). Additionally, FcγRII-deficient mice exhibited increased arthritis, suggesting an inhibitory role of this FcRII in CIA (105) as for the K/BxN STA model (16). Like the CAIA model, both the classical and alternative complement pathways have been shown to be important in the CIA model (108) and, like in the K/BxN STA model (14), C5a and C5aR are key factors in the pathogenesis of the CIA model (107).

The two models share the involvement of autoantibodies, although the specificity of the autoantibodies varies between the two. In the CIA model, the antibodies are directed against CII, while in the K/BxN STA model, the antibodies are anti-G6PI. Moreover, in the CIA model, the autoantibodies predominantly belong to the IgG2a subclass (104), while in the K/BxN STA model, they mainly consist of IgG1 (11). With respect to the involvement of cytokines, TNF (80), IL-1β (80), G-CSF (109), GM-CSF (112), IL-6 (113), and IL-17 (114) are all important drivers in the pathogenesis in CIA, while IL-4 has been shown to mediate a suppressive effect (120). In the K/BxN STA model, it appears that IL-1β (57) and G-CSF (39) are crucial for arthritis progression, TNF (57) and GM-CSF (43) have a partial role, and both IL-6 (57) and IL-4 (115) play no role.

A major practical advantage of the K/BxN STA model over the CIA model is that it induces a robust inflammatory response in a range of different mouse strains with 100% incidence and with a similar onset. As mentioned previously, the CIA model, on the other hand, induces a robust response mainly in susceptible strains, such as DBA1, B10.Q, and B10.QRIII, while in C57BL/6 mice, the incidence and severity are less pronounced with a variable time of onset (101). Moreover, the CIA model runs for 5–6 weeks, while the K/BxN STA model is normally terminated 10–15 days after serum injection. This means that with the K/BxN STA model, an answer to a potential question can be obtained much quicker than what is the case with the CIA model. Furthermore, the K/BxN STA model is independent of an immunostimulatory component, such as CFA, which induces a severe stimulation of the innate immune system.

Another important difference between the models is that the CIA model represents both the priming and effector phases of arthritis and therefore mimics more features of RA than the K/BxN STA model; nonetheless, since the K/BxN STA model only represents the effector phase, one of its advantages is that it can be used to study the biology of this phase. The above discussion indicates that even though the CIA model often is the first choice for a murine model for RA, the K/BxN STA model does offer some advantages and can be a valuable supplement to the CIA model.

## The K/BxN STA Model as a Model for RA

The K/BxN STA model is a valuable tool in understanding the pathogenic mechanisms behind autoantibody-driven arthritis. However, it is essential to consider in what aspects the K/BxN STA model resembles RA and where it differs. The main similarities and differences between the features of the K/BxN STA model and RA are listed in Table 2. When looking at the clinical manifestations and histopathological findings, it is evident that this murine arthritis exhibits many features similar to RA such as leukocyte invasion, synovitis, pannus formation, cartilage and bone destruction, and remodeling of the joint (4). However, the progression of arthritis in the K/BxN STA model is more aggressive developing over a few days, whereas the development of RA disease is a very long process with a peak incidence between 30 and 50 years of age (121). When considering the disease drivers, arthritis in the K/BxN STA model is solely mediated by autoantibodies, while in RA, even though autoantibodies are present and known to participate in the pathogenesis, the disease is additionally driven by other mechanisms involving, for example, CD4 T cells (122). An important role for antibodies in RA is, nevertheless, suggested by the success of anti-CD20 therapy (91). Moreover, autoantibodies of several different specificities have been found in RA patients, including antibodies against citrullinated filaggrin, the Fc portion of IgG [rheumatoid factor (RF)], keratin, chondrocyte gp39, and heat-shock protein 60 (123). Anti-citrullinated filaggrin antibodies have been shown to be useful as a prognostic marker in patients with early RA (124), while in general, the anti-citrullinated protein antibodies (ACPA) are found in 60% of RA patients and are believed to be a critical hallmark in the pathogenesis of RA (125). Moreover, anti-type II collagen antibodies may contribute to disease development even though they are not primary drivers of disease (123). RF is found in 80% of RA patients, and patients with a positive test result for RF in blood have more severe clinical disease and complications than seronegative patients (126, 127), although the pathophysiological role of RF in RA is still unclear. Of note, RF is not present in the K/BxN STA model (126).

**Table 2:** **Similarities and differences between the K/BxN STA and RA models**.

Feature	K/BxN STA model	RA
Histopathological manifestations	• Leukocyte invasion • Synovitis • Pannus formation • Cartilage and bone destruction • Joint remodeling (4)	• Leukocyte invasion • Synovitis • Pannus formation • Cartilage and bone destruction • Joint remodeling (4)
Autoantibodies	Main driver (9)	Present – role unknown (122)
Specificity of autoantibodies	G6PI (2)	• G6PI • Citrullinated filaggrin • Fc portion of IgG (rheumatoid factor) • Keratin • Chondrocyte gp39 • Heat-shock protein • Collagen II (123)
Important immune cells	• Neutrophils (25) • Macrophages (41) • Mast cells? (45, 46)	• Neutrophils • Macrophages • CD4 T cells • B cells • Dendritic cells (55)
Pro-inflammatory cytokines	• TNF (57) • IL-1β (57) • GM-CSF (43) • G-CSF (39) • MIF (83, 84) • IL-17? (62, 63)	• TNF (55) • IL-6 (55) • GM-CSF (44)
Pain	Present and persists after resolution of inflammation (87)	Present and persists after resolutionof joint swelling with anti-inflammatory treatment (85)
Common therapies	• NSAIDs (65) • TNF blockade (57) • Glucocorticoids (128, 129) • Methotrexate (130) • Tacrolimus (131)	• NSAIDs • TNF blockade • Glucocorticoids • Methotrexate • Tacrolimus (66)

Given the essential role of anti-G6PI antibodies in the K/BxN STA model, antibody titers to G6PI have also been investigated in RA patients, however with conflicting results. Initially, it was reported that 64% of RA patients had high levels of anti-G6PI antibodies, which were significantly different from healthy controls (132), but subsequent studies could not reproduce these findings (133–136). A later study suggested that autoantibodies to G6PI were associated with the occurrence of extra-articular complications, such as Felty’s syndrome characterized by neutropenia and an enlarged spleen (137); another study showed that autoantibodies to G6PI were not unique to patients with RA but were found in many patients with inflammatory arthritis (138). These results indicate that G6PI might be one of the several autoantigens able to serve as a target for autoantibodies in RA, but their pathogenic and diagnostic relevance is currently not clear. In this context, G6PI has recently been shown to promote proliferation and to inhibit apoptosis in fibroblast-like synoviocytes from RA patients (139). Despite the uncertain relevance of the different types of autoantibodies, it is likely that, as in the K/BxN STA model, autoantibodies in RA drive the inflammatory response by IC formation and deposition, complement activation as well as activation of Fc receptors on innate immune cells such as neutrophils, macrophages and mast cells (122). However, in contrast to disease progression relying entirely on autoantibodies and their activation of innate immune cells as found in the K/BxN STA model, RA is driven by complex interactions between a range of different cell types from both the innate and the adaptive immune systems, such as T cells, B cells, macrophages, DCs, and neutrophils (55).

With regard to disease mediators, TNF is an important mediator in many, but not all, cases of RA, as shown by the success of TNF blockade in treating the disease. In contrast, neutralizing IL-1 in clinical settings has been shown to have only a limited effect (140). As mentioned, in the K/BxN STA model, IL-1 is absolutely required for arthritis development, and TNF appears to play an important, but partial, role in the disease pathogenesis (57). Additionally, blockade of GM-CSF or its receptor are currently being tested in RA trials with encouraging results (44), and GM-CSF has also been shown to be an important cytokine in the K/BxN STA model (43). The crucial role of macrophages and neutrophils in the K/BxN STA model (25, 41) and the fact that both cell types are found in increased numbers in the inflamed synovia in RA patients (55) suggest that they are also likely important cell types in RA.

As mentioned previously, the most severe symptom frequently reported in RA patients is pain, which can persist after the resolution of joint swelling following anti-inflammatory treatment (85). Arthritis in the K/BxN STA model led to persistent pain with mechanical hypersensitivity, unlike the joint swelling, not returning to baseline but outlasting the inflammation by 2 weeks (86). Moreover, the pain was in the inflammatory phase shown to be sensitive to treatment with NSAIDs and etanercept (TNF blocker) (86), suggesting that the K/BxN STA model might be useful for the exploration of the mechanisms and novel treatment strategies for RA pain.

Current treatment strategies for RA include NSAIDs, glucocorticoids, disease-modifying anti-rheumatic drugs (DMARDs), such as methotrexate, and biologic response modifiers, for example, the blockade of TNF and IL-6 (66). When considering the relevance of the K/BxN STA model as a tool to identify drug candidates with potential therapeutic effect in RA, it is noticeable that several of the approved drugs to treat RA have also been shown to have immunosuppressive effects in the K/BxN STA model. Besides the already mentioned effect of blocking TNF, glucocorticoids, which are widely used in RA, also suppress progression of arthritis (128, 129). Additionally, abrogation of arthritis was obtained with inhibition of COX1 and COX2 by treatment with NSAIDs (65). Finally, the widely used DMARD for RA, methotrexate (130), as well as another DMARD, the calcineurin inhibitor, Tacrolimus (131), had a therapeutic effect in the model. This indicates that the model can have a predictive value in the preclinical screening of drug candidates for RA.

In summary, the K/BxN STA model provides a useful tool for studying certain aspects of RA including autoantibodies, complement, ICs, Fc receptors, and innate cell types, such as neutrophils and macrophages. The increasing focus on autoantibodies and especially ACPA in the pathogenesis in RA potentially raises the relevance of the K/BxN STA model as the one to study how autoantibodies drive autoimmune disease. RA is a heterogeneous disease involving multiple different immunological pathways and accumulation of multiple autoantibody specificities, which differ from patient to patient (66). Therefore, it is not possible to find an animal model that covers all phases and aspects of RA. Thus, as for any animal model, the K/BxN STA model does not encompass all of the features of RA, but mimics several facets of the effector phase. A useful exercise would be to create a “pathogenesis map” outlining the current knowledge of RA (and other types of arthritis) and align the various animal models according to the specific aspect/subset of the disease that each of them reflects. This “map” would facilitate choosing the most appropriate model to address a given question or to study a particular pathway (141, 142). In such a “map,” the K/BxN STA model would be highly relevant as a model to study the effector phase of RA and how autoantibodies drive joint inflammation.

## Conclusion

As an arthritis model, the K/BxN STA model has some obvious advantages. First, it has a rapid onset beginning 2–3 days after transfer of serum with 100% incidence in genetically identical animals. The arthritis is very robust and reproducible, even though minor inter-individual variability can be seen. Second, it can be induced in a wide range of strain backgrounds and therefore also in different KO strains to study the importance of different disease mediators. Furthermore, the model is independent of immunostimulatory components, such as LPS and CFA. Even though G6PI might not be an essential autoantigen in RA, the K/BxN STA model is a useful tool to understand how autoantibodies, in general, drive the progression of arthritis by interacting with different downstream components of the innate immune system. A further advantage of the K/BxN STA model is that it provides an opportunity to study only the effector phase of arthritis without involving the priming phase of the immune response. Finally, the model has also proven useful for the study of arthritic pain. Taken together, these features make the K/BxN STA model a relevant one for RA and, even though not discussed, other arthritides; it is also a potentially valuable tool for the preclinical screening of new therapeutic targets. Although the model has been characterized to a great extent, many aspects of arthritis and pain development, including the roles of regulatory components, different chemokines, and other pro-inflammatory cytokines, are still unknown and need to be addressed.

## Author Contributions

A.D.Christensen participated in the design and wrote the manuscript. CH and A.D.Cook participated in the design and helped draft the manuscript. JH provided intellectual support and helped draft the manuscript. All authors read and approved the final manuscript.

## Conflict of Interest Statement

AC and CH are employees at Novo Nordisk A/S and declare that they have no non-financial conflicts of interest. The remaining coauthors declare that the research was conducted in the absence of any commercial or financial relationships that could be construed as a potential conflict of interest.

## Abbreviations

ACPA, anti-citrullinated protein antibodies; APC, antigen-presenting cell; C5aR, C5a receptor; CAIA, collagen antibody-induced arthritis; CFA, complete Freund’s adjuvant; CGRP, calcitonin-gene-related peptide; CIA, collagen-induced arthritis; CII, type II collagen; COX, cyclooxygenase; DCs, dendritic cells; DMARDs, disease-modifying anti-rheumatic drugs; FcγR, Fcγ receptor; G6PI, glucose-6-phospate isomerase; G-CSF, granulocyte colony-stimulating factor; GM-CSF, granulocyte–macrophage colony-stimulating factor; ICA, immune complex-induced arthritis; ICs, immune complexes; IFA, incomplete Freund’s adjuvant; IFNγ, interferon γ; IgGs, immunoglobulins; IL, interleukin; IRF, interferon regulator factor; IVIG, intravenous immunoglobulin; KO, knockout; LPS, lipopolysaccharide; mAb, monoclonal antibodies; MHC, major histocompatibility complex; MIF, macrophage migration inhibitory factor; MMP, matrix metalloproteinase; MyD88, myeloid differentiation factor 88; NK cells, natural killer cells; NK T cells, natural killer T cells; NOD, non-obese diabetic; NSAIDs, non-steroidal anti-inflammatory drugs; PAMP, pathogen-associated molecular pattern; PGs, prostaglandins; RA, rheumatoid arthritis; RANKL, receptor-activator-of-nuclear-factor-κβ ligand; RF, rheumatoid factor; STA, serum-transfer arthritis; Syk, spleen tyrosine kinase; TCR, T-cell receptor; TLR, Toll-like receptor; TNF, tumor necrosis factor; TGF-β1, transforming growth factor β1; WT, wild-type.
